# An in vitro evaluation of the effect of antimicrobial treatment on bovine mammary microbiota

**DOI:** 10.1038/s41598-024-69273-y

**Published:** 2024-08-07

**Authors:** Anja R. Winther, Aurelie Perrin, Anne O. O. Nordraak, Morten Kjos, Davide Porcellato

**Affiliations:** 1 Faculty of Chemistry, Biotechnology and Food Science, The Norwegian University of Life Sciences, Christian Magnus Falsens Vei 18, 1433 Ås, Norway; 2 Present Address: Institute Agro Dijon, 26 Bd Dr Petitjean, 21079 Dijon, France; 3 Present Address: Norwegian Defence Research Establishment, Kjeller, Norway

**Keywords:** Antimicrobial resistance, Bovine milk microbiota, Mastitis, Sequencing, Antimicrobials, Microbial communities

## Abstract

Antimicrobial-resistant bacteria have been an increasing problem in human medicine and animal husbandry since the introduction of antimicrobials on the market in the 1940s. Over the last decades, efforts to reduce antimicrobial usage in animal husbandry have been shown to limit the development of resistant bacteria. Despite this, antimicrobial-resistant bacteria are still commonly detected and isolated worldwide. In this study, we investigated the presence of antimicrobial-resistant bacteria in bovine milk samples using a multiple approach based on culturing and amplicon sequencing. We first enriched milk samples obtained aseptically from bovine udders in the presence of two antimicrobials commonly used to treat mastitis and then described the resistant microbiota by amplicon sequencing and isolate characterization. Our results show that several commensal species and mastitis pathogens harbor antimicrobial resistance and dominate the enriched microbiota in milk in presence of antimicrobial agents. The use of the two different antimicrobials selected for different bacterial taxa and affected the overall microbial composition. These results provide new information on how different antimicrobials can shape the microbiota which is able to survive and reestablish in the udder and point to the fact that antimicrobial resistance is widely spread also in commensal species.

## Introduction

Mastitis is an inflammation of the udder, often caused by bacteria, with high cost to the dairy industry and a large impact on animal welfare^1,2^. Bacteria enter the udder through the teat canal during milking or from the cow’s environment, such as bedding materials^1^. Mastitis can be classified as clinical or subclinical. The clinical cases display signs such as fever, redness, swelling, and pain in addition to an increase of the somatic cell count (SCC) in the milk from 10,000 to 100,000 cells/mL to several million cells/mL. The increased number of somatic cells is due to immune cells that migrate to the udder lumen to fight off the infection and increased shedding of the udder epithelial cells. Subclinical cases are not associated with the abovementioned signs and present a SCC of 100,000 to several million cells/mL depending on the pathogen^3–6^. An essential part of combating mastitis is through antimicrobial treatment. However, several studies show that antimicrobial agents have a limited impact on the pathogenic bacteria causing the infection, mainly when the infectious bacterium is Gram-negative^7–9^.

Gram-positive bacteria are the most frequent findings in mastitis in Norway^10^, and penicillin is currently the first choice for treating bacterial mastitis^11^. In cases where penicillin resistance is detected, other antimicrobial agents, such as a combination of amoxicillin and clavulanic acid (AMC), are available^12^. Penicillin and amoxicillin are β-lactams targeting bacterial cell wall synthesis and are widely used in veterinary medicine^13^. Amoxicillin is typically administered with clavulanic acid, an inhibitor of β-lactamases, enzymes that a bacterium can produce that binds and cleaves β-lactams so that they are no longer harmful to the cell. The treatment for mastitis is commonly a combination of intramammary and systemic injections of the chosen antimicrobial agent in combination with optimal cow comfort and supportive therapy such as fluid therapy^14^. Dry cow therapy is another way the bacteria in the udder are exposed to antimicrobials^15^. As the cow has a risk of contracting an infectious bacteria causing mastitis during the dry period, a common practice has been to treat the cow with intramammary antimicrobial agents, often cloxacillin, at dry-off to reduce the risk of developing mastitis during the next lactation cycle^1^. Preventive treatment at dry-off has not been practiced in Norway, but selective dry cow therapy is increasingly used in Norway and other Nordic countries. This means that only cows with a high SCC and the presence of typical mastitis pathogens are treated before the dry period^14^.

Inappropriate use and overuse of antimicrobial agents are the main reasons for developing and spreading antimicrobial-resistant bacteria over the last decades. The recent focus on reducing antimicrobial agents in food-producing animals has shown some positive effects. In 2021, the population-weighted mean antimicrobial use in food-producing animals was lower than in humans for the first time^16^. This starkly contrasts with 2017, when 73% of all antimicrobials used worldwide were in animals^17^. Penicillin resistance amongst bacteria isolated from cases of mastitis in Norway is low^10,18^. Even though the usage of antimicrobial agents in Norway has been reduced over the last few decades, we know resistance is a fast-spreading issue worldwide and is considered the next pandemic. The current method used to isolate resistant bacteria in the laboratory is by cultivation on agar. However, it is well known that not all bacteria can grow under these standard laboratory conditions, as they are often optimized for the growth of the most common mastitis pathogens^1^. High-throughput sequencing of 16S rRNA genes has already been used on bovine milk samples to show the presence of diverse bacterial groups that often go undetected with traditional cultivation methods^19,20^.

In this study, we aimed to investigate whether resistant isolates were present as part of the mammary microbiome in dairy cows and how the milk microbial composition might be affected by treatment with antimicrobial agents. To answer this question, we selected cows from a farm that has historically had a healthy herd with minimal usage of antimicrobials, in vitro enriched milk samples taken aseptically from healthy udders in milk containing penicillin G or AMC and studied the surviving bacteria. In addition to the traditional cultivation method to isolate and identify antimicrobial-resistant bacteria, we also used amplicon sequencing to investigate which bacteria survived and tolerated the antimicrobial treatment. Utilizing this additional technique allowed us also to detect the genera that did not grow under standard laboratory conditions.

## Results

### Milk samples and study design

The milk samples used in this study were bovine quarter samples collected from Norwegian Red cows for a study characterizing the microbiota in milk samples from healthy udders with high (> 100,000 cells/mL) and low (< 100,000 cells/mL) somatic cell count (SCC)^3^. Thus, in these samples, the heightened SCC was not from quarters with clinical mastitis but could indicate subclinical mastitis and showed that quarters with a high SCC generally displayed a less stable and less diverse microbiota than quarters with a low SCC. In addition to the 45 samples from the published study, three fresh samples from three cows from the same farm were included in the current study. One of these had a low SCC (< 100,000 cells/mL), and two had high SCC (> 100,000 cells/mL). The experiment comprised 48 samples, 25 with a high SCC (H01-H25) and 23 with a low SCC (L01-L23). Once the milk samples were thawed, 0.1 mL of each sample was plated on TSA blood agar and incubated overnight (T0). After incubation, the number of colonies and the different morphologies were recorded. The results of this analysis can be found in Table 1. Milk from each sample was then inoculated in UHT milk with penicillin G or the AMC combination. To monitor the growth of the bacteria in the milk over the next three days, 0.1 mL of the milk cultures were plated on TSA blood agar daily. In the cases where an increasing number of colonies were detected over the three days, indicating that the bacteria could grow in the presence of penicillin G or AMC, colonies were inoculated in liquid BHI and kept for further analysis. The concentrations used for penicillin G (0.125 µg/mL) and AMC (0.25 µg/mL) were based on EUCAST breakpoint tables and literature^21–23^. We found bacteria that were able to tolerate one or both antimicrobial agents in 35 of the 48 samples. In total, 92% (23 of 25) of the samples with high SCC harbored resistant bacteria, and 52% (12 of 23) with low SCC harbored resistant bacteria. As amoxicillin is considered a broader spectrum antimicrobial compared to penicillin^24^, it was surprising that all samples except one where we isolated resistant bacteria had one or more isolates resistant to AMC, while only 20 of the 35 samples with resistant bacteria had isolates resistant to penicillin G.

**Table 1:** Overview of isolates resistant to PEN (n = 29) or AMC (n = 55).

Sample	Colonies T0	PEN MALDI-TOF MS identification	AMC MALDI-TOF MS identification
H01	800 (3)	*Enterococcus durans, Escherichia coli*	Not identified
H02	180 (4)	*Enterococcus faecalis*	*Staphylococcus aureus, Enterococcus faecalis*
H03	55 (2)	*Enhydrobacter aerosaccus/Moraxella osloensis, Micrococcus luteus*	*Corynebacterium amycolatum*
H04	20 (1)	No resistant isolate found	Not identified
H05	14 (3)	*Micrococcus luteus, Enhydrobacter aerosaccus/Moraxella osloensis*	*Staphylococcus chromogenes*
H06	95 (3)	*Staphylococcus epidermidis*	*Staphylococcus epidermidis*
H07	5 (3)	No resistant isolate found	No resistant isolate found
H08	136 (2)	*Staphylococcus aureus*	*Staphylococcus aureus, Streptococcus dysgalactiae* spp.* equisimilis/dysgalactiae, Staphylococcus simulans*
H09	461 (2)	No resistant isolate found	*Staphylococcus epidermidis, Staphylococcus hominis*
H10	401 (2)	*Staphylococcus epidermidis, Corynebacterium amycolatum*	*Staphylococcus epidermidis, Staphylococcus haemolyticus*
H11	51 (2)	Not identified	*Staphylococcus chromogenes*
H12	15 (1)	No resistant isolate found	*Staphylococcus chromogenes, Staphylococcus saprophyticus*
H13	2 (1)	*Corynebacterium bovis*	*Staphylococcus chromogenes, Staphylococcus xylosus, Bacillus subtilis, Staphylococcus epidermidis*
H14	21 (4)	No resistant isolate found	*Micrococcus luteus, Staphylococcus saprophyticus*
H15	233 (2)	*Staphylococcus epidermidis*	*Staphylococcus epidermidis*
H16	0	Not identified	No resistant isolate found
H17	1 (1)	No resistant isolate found	*Staphylococus saprophyticus*
H18	112 (1)	No resistant isolate found	*Corynebacterium bovis*
H19	1 (1)	No resistant isolate found	*Staphylococcus cohnii* spp.* cohnii*
H20	2 (2)	No resistant isolate found	No resistant isolate found
H21	256 (3)	No resistant isolate found	*Staphylococcus xylosus*
H22	112 (2)	No resistant isolate found	*Staphylococcus chromogenes*
H23	0	*Staphylococcus epidermidis*	*Staphylococcus epidermidis, Staphylococcus hominis*
H24	174 (1)	*Staphylococcus epidermidis, Acinetobacter radioresistens*	*Staphylococcus epidermidis, Acinetobacter baumannii*
H25	11 (2)	*Weissella paramesenteroides, Kocuria rhizophila, Staphylococcus epidermidis*	*Staphylococcus haemolyticus, Staphylococcus epidermidis*
L01	49 (3)	*Enterococcus faecalis*	*Escherichia coli, Stenotrophomonas maltophilia*
L02	1 (1)	No resistant isolate found	Not identified
L03	30 (1)	*Enterococcus faecium, Enterococcus faecalis*	*Macrococcus caseolyticus, Enterococcus faecalis*
L04	0	No resistant isolate found	No resistant isolate found
L05	0	*Escherichia coli*	Not identified
L06	1 (1)	No resistant isolate found	No resistant isolate found
L07	0	No resistant isolate found	No resistant isolate found
L08	0	No resistant isolate found	*Streptococcus parasanguinis, Staphylococcus cohnii* spp.* cohnii, Staphylococcus hominis, Staphylococcus xylosus*
L09	0	No resistant isolate found	No resistant isolate found
L10	1 (1)	*Micrococcus luteus*	Not identified
L11	1 (1)	No resistant isolate found	No resistant isolate found
L12	12 (1)	No resistant isolate found	No resistant isolate found
L13	0	No resistant isolate found	*Micrococcus luteus, Staphylococcus xylosus*
L14	0	No resistant isolate found	No resistant isolate found
L15	1 (1)	No resistant isolate found	No resistant isolate found
L16	0	*Staphylococcus epidermidis*	*Staphylococcus epidermidis*
L17	4 (1)	No resistant isolate found	*Staphylococcus epidermidis, Bacillus subtilis*
L18	1 (1)	No resistant isolate found	No resistant isolate found
L19	2 (1)	No resistant isolate found	No resistant isolate found
L20	0	No resistant isolate found	*Staphylococcus epidermidis*
L21	0	*Staphylococcus epidermidis, Staphylococcus capitis*	*Staphylococcus epidermidis, Pediococcus pentosaceus*
L22	1 (1)	No resistant isolate found	No resistant isolate found
L23	0	No resistant isolate found	Not identified

The isolates were identified with MALDI-TOF MS, utilizing the VITEK MS Expanded V3.2 Database. Results with a confidence value greater than 98% were categorized as identified. T0 indicates the number of colonies observed when culturing the milk samples on blood agar before antimicrobial treatment. The number in parenthesis indicates the number of different morphologies of the colonies.

*AMC* amoxicillin/clavulanic acid, *PEN* penicillin G.

### Identification of the resistant isolates using MALDI-TOF MS

To identify the bacteria that survived the antimicrobial treatment, matrix-assisted laser desorption/ionization time-of-flight mass spectrometry (MALDI-TOF MS) was utilized. Table 1 displays an overview of the bacteria isolated from the two treatments. Seventy-six isolates were identified with MALDI-TOF MS. Six isolates (7%) were not identified. The isolates were found to be both typical mastitis pathogens such as *Staphylococcus aureus*, *E. coli*, *Enterococcus faecalis*, and *Corynebacterium bovis*, but also *S. epidermidis*, *S. chromogenes* and *Streptococcus parasanguinis* which might be considered to be part of the natural microbiota^1,25^. As evident in Table 1, many of the bacteria identified as resistant to AMC were staphylococci (69%). These results show a range of bacteria with acquired or innate resistance to both penicillin G and AMC present in the bovine udder.

### The minimal inhibitory concentration of the isolates

**Figure 1:**
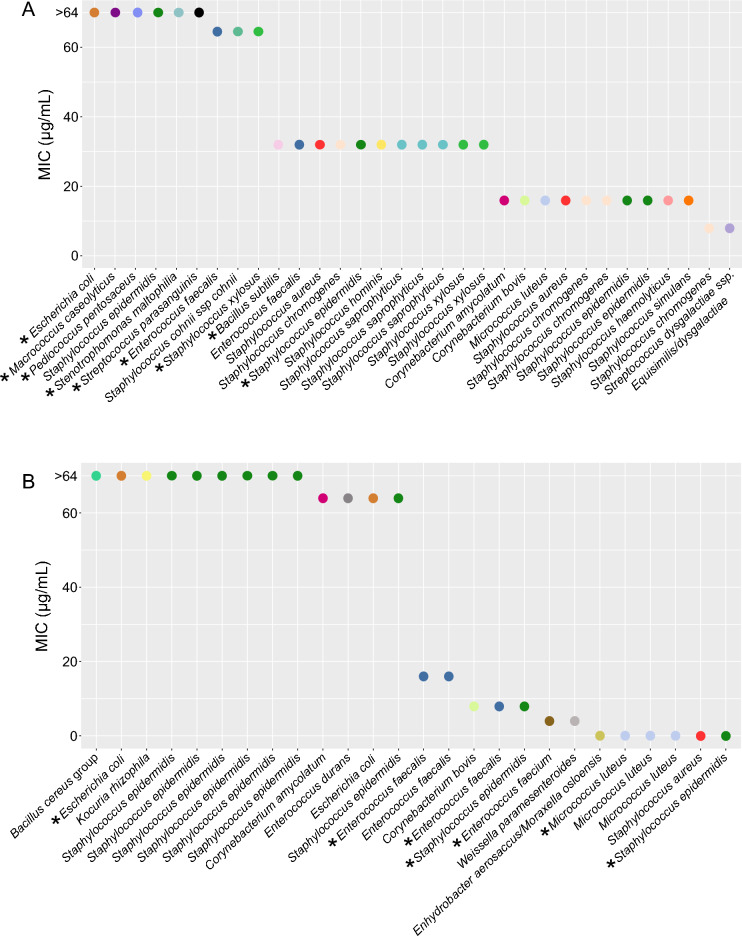
Scatterplot of the minimal inhibitory concentration (MIC). (**A**) MIC (µg/mL) for isolates resistant to AMC (**B**) MIC (µg/mL) for isolates resistant to PEN. Species are separated with different colors in the plots. The asterisk indicates that the specie was isolated from a L sample. Some isolates proliferated in all the concentrations tested in the assay. The MIC was registered as > 64 µg/mL in those cases. The MIC for each isolate was determined for the antimicrobial agent the isolate initially proliferated in. The MIC value was determined as the lowest concentration of the antimicrobial agent required to completely inhibit bacterial growth after 20 h of incubation. *AMC* amoxicillin/clavulanic acid, *PEN* penicillin G.

Milk contains a range of proteins, fat and other components that might influence the effect of the antimicrobial agents. It was, therefore, necessary to investigate the MIC of the 76 isolates identified with matrix-assisted laser desorption/ionization time-of-flight mass spectrometry (MALDI-TOF MS) under laboratory conditions. For this purpose, the broth microdilution method recommended by the European Committee on Antimicrobial Susceptibility Testing (EUCAST) was used^21^. The MIC value for each isolate was determined for the antimicrobial agent (penicillin G or AMC) in which the isolate initially proliferated. A 96-well plate with a two-fold dilution series of either penicillin G or AMC was used, and the MIC was recorded as the lowest concentration of the antimicrobial agent that completely inhibited bacterial growth after 20 hours^26,27^. Some isolates (3%) did not grow under the conditions in the plate reader and were excluded from the analysis. The results of the MIC assays are displayed in Fig. 1. Supplemental Table S1 contains the full overview of the MIC values for all isolates tested. The AMC combination assay showed MIC values ranging from 8 to > 64 µg/mL. The MIC for penicillin G varied from the isolates being able to tolerate low concentrations of penicillin G (< 0.06 µg/mL) up to high concentrations (> 64 µg/mL). *S. epidermidis* was particularly interesting. Seven out of nine isolates tested against penicillin G had a MIC at or above 64 µg/mL. The susceptibility breakpoint for penicillin G for *S. aureus* is ≤ 0.125 µg/mL, meaning that the *S. aureus* (sample H08) is considered susceptible^21^. Likely, bacteria that were determined to be susceptible to penicillin G in the MIC assay survived in milk with higher concentrations of penicillin G due to the protective effect of milk on the bacteria from the action of the antimicrobial agent.

### Identification of resistant microbiota using amplicon sequencing

**Figure 2:**
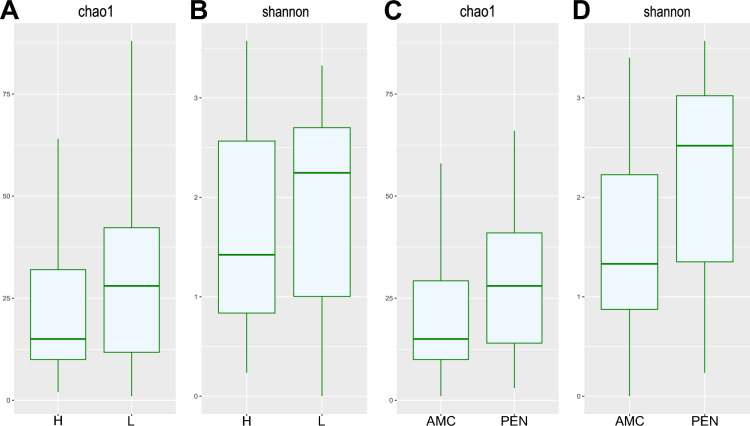
Alpha diversity measurements (chao1 index and shannon diversity) based on the 16S rRNA data. (**A and B**) compare the two diversity measurements between H (SCC > 100,000 cells/mL) and L (SCC < 100,000 cells/mL) samples, while (**C and D**) compare the diversity measurements between cultures grown with PEN and the AMC combination. The difference in alpha diversity between the H and L samples were not significant (p > 0.05). The difference in alpha diversity between the two treatments were significant (p < 0.05). *AMC* amoxicillin/clavulanic acid, *PEN* penicillin G.

In addition to isolating and identifying the resistant bacteria on the agar plates, a culture-independent analysis was performed to see the overall microbial population that was enriched in milk in the presence of both antimicrobials. This was also done to identify bacteria that potentially survived the antimicrobial treatment but did not grow under our standard laboratory culturing conditions. To make sure we only amplified the 16S rRNA genes from bacteria that survived in the milk with the antimicrobial agents, we treated the samples with propidium monoazide (PMAxx) prior to amplification. This is a nucleic acid intercalating dye that is able to permeate the cell membrane of dead cells and, when activated by photolysis, covalently binds DNA and inhibits amplification by PCR^28^. We chose the samples where we detected resistant isolates with the traditional cultivation method and performed 16S rRNA sequencing on these samples. The alpha diversity of the amplicon data on genus level was measured as species relative abundance emphasizing species richness estimation (chao1 index, Fig. 2A,C) and evenness and dominance (Shannon diversity, Fig. 2B,D). The average chao1 index was 22.1 for the H samples (SCC > 100,000 cells/mL) and 31.3 for the L samples (SCC < 100,000 cells/mL). The average Shannon diversity was 1.6 for the H samples and 1.9 for the L samples. Statistical testing with the Kruskal-Wallis rank sum test revealed that the difference between the two groups was not significant (p > 0.05). The difference in alpha diversity between the two groups of antimicrobial agents, on the other hand, was significant (p < 0.05). The average chao1 index for samples grown with AMC was 22.0 and for samples grown with penicillin G was 30.2. The average Shannon diversity was 1.5 for samples grown with AMC and 2.18 for samples grown with penicillin G.

**Figure 3:**
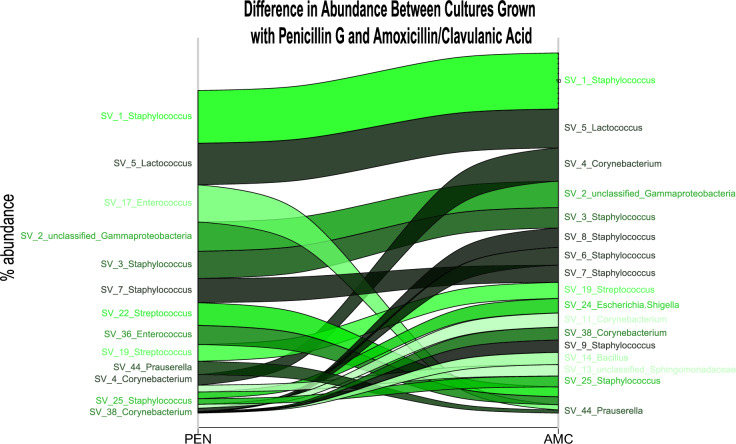
An alluvial plot of the relative abundance of the 20 most abundant sequence variants (SVs) proliferating in milk containing the AMC combination and penicillin G. The plot was made using all the 48 milk sample sequencing data results. *AMC* amoxicillin/clavulanic acid, *PEN* penicillin G.

A comparison of the effect of the antimicrobial agents on the ability of the enriched microbiota to grow in milk was done by summing up all the relative abundance from the samples within the two antimicrobial groups. Interestingly, some patterns were shown when the data were analyzed at the sequence variance (SV) level (Fig. 3). While some staphylococcal SVs remained more or less the same between the two treatments (e.g., SV_1, SV_3, SV_7, all representing *S. epidermidis*), some displayed a more noticeable change. In particular, SVs 6 and 8 were more abundant in the AMC cultures. A BLAST search revealed that these SVs were *S. xylosus* and *S. chromogenes*, respectively. Additional SVs that were more abundant in the AMC treated cultures included SV_4 and SV_11 corresponding to *Corynebacterium bovis* and SV_38 corresponding to *C. amycolatum*. The SVs corresponding to *Enterococcus* responded differently to the two antimicrobial treatments. SV_17 (*E. hirae*/*E. faecium*) and had a higher abundance after penicillin G treatment, while SV_36 (*E. faecalis*) had a higher abundance after AMC treatment. The SVs corresponding to *Streptococcus* are interesting as they are considered mastitis pathogens and how they respond to antimicrobials will affect the recovery of the cow after an infection. SV_19 displayed a stable abundance in the two treatments, while SV_22 had a reduced abundance in the AMC-treated samples compared to the penicillin G-treated samples. Taken together, this analysis shows that the AMC combination negatively affects the general microbiota in the milk, leaving *Staphylococcus* and *Corynebacterium* to make up the main abundance in these samples. This is also reflected in Table 1, where 69% of the isolates in samples treated with AMC were *Staphylococcus.* Also worth noting is that SV_5 (*Lactococcus lactis*/ *L. cremoris*) was not affected, or was affected equally, by the two treatments. *Lactococcus* species are important starter cultures in dairy fermentation^29^, and live suspensions of *L. lactis* has shown potential as an alternative treatment for antimicrobials in mastitis infections^30^.

**Figure 4:**
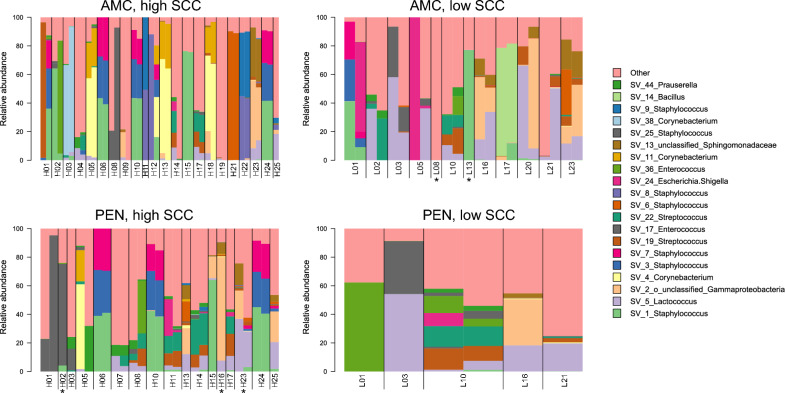
The relative abundance of the 20 most abundant genera plotted for each sample. The two bars for each sample represent day 1 and day 3 of antimicrobial treatment. The 16S rRNA sequencing data were rarefied at 1500 sequences and those samples with less sequences were omitted from the analysis. When only one bar was displayed, the other day had too few sequences to be included in the analysis. This was often day 3. The asterisk labels the instances where day 1 was omitted. High SCC refers to the samples with more than 100,000 somatic cells/mL, while low SCC refers to the samples with less than 100,000 somatic cells/mL. *AMC* amoxicillin/clavulanic acid, *PEN* penicillin G.

To follow the longitudinal development of growth in the two treatments in the high and low SCC milk, the relative abundance of SVs based on the 16S rRNA sequencing data after 1 and 3 days of incubation with antimicrobial agents is plotted in Fig. 4. It is evident from the bar plots in Fig. 4 that the samples from quarters with a high SCC contain a larger diversity of resistant bacteria compared to the samples from quarters with a low SCC. In some samples, the same bacterial species seems to harbor resistance against both AMC and penicillin G, such as the staphylococcal SVs SV_1, SV_3 and SV_7 in samples H06, H10 and H24. This is also reflected in the alluvial plot in Fig. 3. SV_5 (*Lactococcus)*, although found in low abundance, appears in several samples in both treatments. Some samples harbored species with treatment-dependent resistance patterns. This includes sample H01, which on day 3 displayed a high abundance of SV_1, SV_3, and SV_7 (*Staphylococcus*) resistant to AMC. Still, these staphylococci were absent when the sample was treated with penicillin G. Here, SV_17 (*Enterococcus*) was most abundant. The same was true for sample H08, which contained a high amount of SV_25 (*Staphylococcus*) after three days of AMC treatment, while after treatment with penicillin G SV_19 (*Staphylococcus*), SV_22 (*Streptococcus*) and SV_36 (*Enterococcus*) had a high abundance. In the samples taken from quarters with a low SCC the general trend was a lower diversity of resistant bacteria, specifically for the samples treated with penicillin G. The low SCC samples also contained a higher abundance of *Lactococcus* than the high SCC samples. This was most likely due to the lower count of resistant bacteria in these samples, allowing the *Lactococcus* to take up a more significant part of the relative abundance.

## Discussion

In the literature, a common way to detect antimicrobial-resistant bacteria in bovine milk samples has been to plate the samples on agar, identify the isolates, and then test for antimicrobial resistance^22,23,31–35^. However, it is well known that not all bacteria found in bovine milk can grow with the traditional cultivation methods we use today. In this study, we wanted to use a different approach (i.e., 16S amplicon sequencing of antimicrobial-treated milk) to complement the traditional cultivation-based method. While the exact prevalence of resistance was not determined in this work, our investigation gives new insights into which bacteria survive antimicrobial treatment and demonstrates the power of using non-culture methods to monitor the presence of antimicrobial-resistant or non-susceptible bacteria in bovine milk samples. To isolate bacteria resistant to the commonly used penicillin G and AMC, we enriched milk from bovine quarters with high and low SCC in UHT milk containing penicillin G (0.125 µg/mL) or AMC (0.25 µg/mL). The concentrations used were based on the EUCAST breakpoints and, as the breakpoints are based on bacterial strains isolated from humans, literature regarding resistant strains isolated from bovine milk^21–23^. The growth of bacteria in the milk, or lack thereof, was followed by plating on blood agar daily. The bacteria that survived the treatment were identified with MALDI-TOF MS, and the MIC was determined using the standard broth microdilution method. For the detection of resistant bacteria that did not grow on blood agar and to see the effect of the antimicrobial agents on the growth of bacteria, 16S rRNA sequencing was used. Total bacteria were isolated from day 1 and day 3 of incubation with penicillin G or AMC, and all viable bacteria present in the samples were identified with sequencing. Comparing the results of resistant bacteria isolated with the traditional plating (Table 1) and 16S rRNA sequencing (Fig. 4) showed that the traditional cultivation methods can identify the most abundant species that grew with the two treatments, except *Lactococcus*.

Altogether, we identified a higher diversity in bacteria resistant to penicillin G than AMC. This is expected, given that AMC has a broader inhibitory spectrum than penicillin G. Still, we cannot exclude the interaction between the antimicrobial agents and the milk which might have had a different impact on the activity of penicillin G compared to AMC. Milk is a complex medium with various components that could bind to and/or sequester the antimicrobial agent and thereby reduce the efficiency. It has been shown that β-lactams bind to milk proteins, which might decrease the antimicrobial activity and spectrum^36^. However, whether penicillin G is affected differently than AMC in this respect is unknown. In addition, a significant proportion of the milk bacteria is associated with fat globules^37–39^. It could be speculated that the fat globules serve as partial protection against the antimicrobial agent so that the bacteria are not exposed to the concentration required to inhibit or kill. The higher alpha diversity observed for the L samples in Fig. 2A,B, despite not being significant, could be explained by the origin of the samples. The L samples are from udders with a SCC below 100,000 cells/mL and were shown to have a significantly high alpha diversity compared to samples taken from udders with a high SCC (H samples) in our previous study^3^. It is reasonable to assume that having a more diverse composition of bacteria in these samples would result in a more diverse community resistant to AMC and penicillin G. As amoxicillin is considered a broader spectrum antimicrobial compared to penicillin G, the higher diversity of the community after treatment with penicillin G is in line with the expected result. This also aligns with the results of the MALDI-TOF MS where we observed that a major part (69%) of the isolates resistant to AMC were staphylococci, while the isolates resistant to penicillin G were a more diverse group.

The identification of resistant bacteria from both culture-dependent and culture-independent analysis shows that the commensal species, such as *S. chromogenes* and *S. parasanguinis*, can harbor innate resistance to antimicrobial agents and/or develop resistance to the antimicrobials they are exposed to. Non-aureus staphylococci (NAS), such as *S. chromogenes*, are commonly found in bovine milk and cause subclinical mastitis^40,41^. It is a well-known phenomenon that these NAS can carry multiple resistance genes such as *ampA*, *ermC*, *blaZ*, and *tetK* making them resistant to ampicillin, erythromycin, penicillin and tetracycline^42,43^. Multi-resistance in these species is of particular concern as they are potentially pathogenic for humans through enterotoxin production^44–46^. It is also apparent from the results that there is a range of bacteria (labeled “other” in Fig. 4) in the milk that could tolerate the antimicrobial agents used in the study or were somehow protected from these antimicrobials by the properties of the milk. Although not surprising, this is potentially concerning as these commensal and less abundant species can become a pool of resistance genes that the pathogens can acquire when they breach the udder defenses, making an infection such as mastitis complicated to treat. If commensals and pathogens alike harbor resistance to antimicrobial agents and are protected against their activity by the milk, it might be necessary to evaluate traditional treatments against mastitis. Alternatives to the commonly used antimicrobials have been reported, including bacteriophages, antimicrobial peptides, and herbal antimicrobial substances^47–49^.

During the treatment of mastitis, most bacteria in the udder are exposed to and affected by the antimicrobial agent administered. The species that are less or not at all affected by the treatment have an ecological advantage to reestablish and dominate the microbial community and potentially affect the general udder health^50,51^. To date, no clinical studies have proven that the milk microbiota is beneficial for the cow’s protection against mastitis or the general health of the udder. There are, however, several studies reporting on the correlation between the bacteria commonly found in milk samples. Some commensal species can prevent the growth of mastitis pathogens by producing antimicrobial materials^52–54^. In contrast, others can modulate the host production of anti-inflammatory cytokines^55^. Some studies have shown that dysbiosis in the udder microbiota caused by an intramammary infection or cephalosporin treatment is reversed after the infection ends or the antimicrobial is cleared from the udder^1,50^. The rate of clearance and how fast the initial microbial community is reestablished are also greatly affected by the genetic composition of the cow^1^. However, the microbial community in the udder goes through a disruption during an infection and the following treatment, and the species less affected will be necessary for shaping the microbial community in the udder, at least for a while.

No *Lactococcus* were isolated with the traditional culture-dependent methods, while the 16S rRNA sequencing detected *Lactococcus* (SV_5) as part of the 20 most abundant genera. In the literature, *Lactococcus* is often isolated from bovine milk on MRS agar on 30 ℃ and not the blood TSA agar and 37 ℃ used in this project^56,57^. This shows that the chosen culturing method dramatically affects the isolates one finds in such experiments. In Fig. 4, SV_5 can be found in several samples from both high and low SCC quarters. The alluvial plot in Fig. 3 reveals that the relative abundance of the *Lactococcus* does not seem to be affected differently by the two treatments. A BLAST search revealed that SV_5 is *L. lactis* or *L. cremoris*. These are important species in dairy starter cultures, can be important in probiotics and *L. lactis* can produce bacteriocins that could potentially be beneficial for mastitis control^30,58^. As *L. lactis* has a positive effect on preventing mastitis development in the udder and dairy production, it seems valuable to have it survive antimicrobial treatment. As no *Lactococcus* were isolated using the culture-dependent method, we cannot test the MIC for this genus. Whether *Lactococcus* was resistant to penicillin G and AMC or protected by the milk is hence unknown. In the literature, *L. lactis* displays low levels of resistance to penicillin G and AMC^59,60^. Still, having this beneficial genus surviving in the udder despite the presence of antimicrobial agents could be beneficial for reestablishing the commensal microbiota after an infection.

*S. epidermidis* is, together with the other NAS species, a common cause of subclinical mastitis. As the NAS species are frequently isolated from bovine milk samples and considered to be opportunistic pathogens, *S. epidermidis* might be a common part of the milk microbiome^51^. It has been shown that *S. epidermidis* can produce bacteriocins that efficiently inhibit some *S. agalactiae* strains isolated from bovine mastitis^61^. This would make this species beneficial in the udder after antimicrobial treatment to modulate the growth of pathogens. SV_1, SV_3 and SV_7 all corresponded to *S. epidermidis,* and it was particularly interesting that these were equally affected by the two treatments. It could thus be speculated that these have a selective advantage after antimicrobial treatment and are likely part of shaping the udder microbiota after an infection.

As observed for *Lactococcus*, not all species will grow under the conditions in the laboratory. Culturing milk samples on blood agar before antimicrobial treatment (T0, Table 1) often resulted in growth from milk samples with high SCC and no or little growth from samples with low SCC. However, some samples displayed the opposite trend. It is possible that for samples with low SCC, the colonies that grew on blood agar were part of the contamination from the barn environment or teat skin. Even though this is unfortunate, finding antimicrobial resistance amongst bacteria that are part of the cow’s environment is interesting. These species can gain access to the udder and cause an infection, or their resistance genes can be transferred to pathogens through horizontal gene transfer. High SCC is not only an indication of bacterial infection but can also result from viral or yeast infections or trauma to the udder^1^. This can explain the culture negative results from samples with high SCC.

We detected several species of *Staphylococcus* in the AMC-treated cultures. In AMC, clavulanic acid acts as a β-lactamase inhibitor, allowing amoxicillin to retain its activity against β-lactamase expressing strains, one of staphylococci’s main β-lactam resistance mechanisms. Another major resistance mechanism against β-lactams in this family is through target modulation. These strains have been able to acquire a gene (*mecA*) encoding an alternative penicillin-binding protein (PBP), i.e. one of the enzymes responsible for building the cell wall and the target of β-lactams^62^. This means that the staphylococci do not necessarily utilize β-lactamases (which are inhibited by clavulanic acid) to confer β-lactam resistance, and clavulanic acid would thus not have the enhancing effect when administered with amoxicillin against such strains^63^. Furthermore, there is also evidence for the presence of so-called *mec-independent* oxacillin non-susceptible *S. aureus* (MIONSA) or borderline oxacillin resistance *S. aureus* (BORSA), which display phenotypic β-lactam resistance without the presence of β-lactamases or *mecA*^64–66^. In these cases, the non-susceptibility is normally due to mutations affecting cell wall synthesis and metabolism. We did not investigate the molecular mechanism underlying the non-susceptibility to β-lactams in the current work, but this would be of interest for future studies.

Our results show that despite being part of a healthy herd with little antimicrobial treatment, resistance against the commonly used penicillin G and AMC still occurs among strains in the mammary microbiota. Both methods used to identify resistant bacteria suggested that the major player that might have a selective advantage in reestablishing itself in the udder after antimicrobial treatment is *S. epidermidis* (SV_1, SV_3, and SV_7). The culture-independent method also included *Lactococcus* (SV_5) in this group. The study revealed that more knowledge on the effect of other antimicrobial agents and in vivo experiments are necessary to confirm these results.

## Materials and methods

### Study animals and sample collection

The milk samples used in this study were quarter samples collected from Norwegian Red cows selected from “The Livestock Production Research Centre” at the Norwegian University of Life Sciences with regards to a different project^3^. Left over milk thawed from − 80 ℃ were used in the current study. In addition, three fresh samples were taken from three different cows from the same farm. All samples included in the study were taken from healthy animals and grouped into “high” (H) or “low” (L) based on SCC below or above 100,000 cells/mL on quarter level. The total amount of samples processed were 48. The farm operates under the regulations of the Norwegian Food Safety Authority regarding food production and animal care. Permission for sample collection and use of information regarding the samples was given by the farm owners. No invasive procedures were used in this study. The cattle enrolled in the study were housed in freestalls with cubicles containing bedding materials of rubber mats with raw wood chips. Their diet consisted of silage, continuously available, and supplemented with pelleted feed based on milk production of the individual cow. The herd level SCC remained consistent from 2018 to 2023, fluctuating between a minimum of 91,000 cells/mL and a maximum of 124,000 cells/mL. Throughout this period, the herd experienced a low incidence of mastitis cases, ranging from 0.077 to 0.211. This calculation is derived by dividing the number of mastitis treatments with at least 4 days in between by the total number of year cows in the herd. Samples were collected following the “Procedure for Collecting Milk Samples” of the National Mastitis Council (NMC, www.nmconline.org) at the end of the regular milking routine as previously described^51^. The milking apparatus was removed, the teats were washed with iodine and then alcohol, and 200 mL of milk were collected manually in sterile glass bottles. All the operators changed gloves between samples. The samples were kept on ice and immediately transported to the laboratory, aliquoted into 50 mL falcon tubes, and stored at − 80 ℃.

### Procedure used to screen milk for antimicrobial resistant isolates

The antimicrobial agent penicillin G and the antimicrobial agent/β-lactamase inhibitor combination amoxicillin/clavulanic acid (AMC) were chosen for this project as they are clinically relevant drugs^67–69^. The concentrations utilized were based on literature and EUCAST standards and were 0.125 µg/mL (penicillin G) and 0.25 µg/mL (amoxicillin/clavulanic acid)^21–23^. Upon arrival in the laboratory, 100 µl of each milk sample were plated on TSA blood agar plates (ThermoFischer Scientific, Massachusetts, United States) and incubated at 37 ℃ overnight (T0). The number of colonies on each plate and the different types of morphology were recorded. To screen for bacteria resistant to penicillin G or AMC, one mL of each milk sample was inoculated in 9 mL of UHT milk in six parallels. Three contained penicillin G with the concentration indicated above and three contained AMC. The three parallels with each antimicrobial agent were incubated at 37 ℃ for one, two, and three days. After each incubation period was finalized, 100 µl of each parallel was plated on TSA blood agar plates. When the number of colonies on TSA blood agar plates increased from day one to day three, isolates of different morphology were picked, grown in BHI broth (Oxoid), and kept for further analysis. The remaining milk from each parallel was prepared for DNA extraction and amplicon sequencing as described below.

### DNA extraction and amplicon sequencing

For the identification of resistant bacteria that grew in the milk but did not appear on the blood agar plates, 16S amplicon sequencing was utilized as described previously^51^. In brief, the milk samples were centrifuged at 8000 × *g* for 5 min, and the fat layer was removed with a sterile cotton swab. After removal of the supernatant, the pellet was washed twice with 2% citrate water, dissolved in 500 µl H_2_O, and treated with PMAxx (Biotium) according to the manufacturer’s protocol. Finally, the DNA was extracted from each pellet using the DNeasy PowerFood Microbial Kit (Qiagen, Düsseldorf, Germany) starting from step 3 in the detailed protocol of DNeasy Powerfood Microbial Kit Handbook. To prepare the library for amplicon sequencing the V3 and V4 regions of the 16S rRNA gene were amplified with the primers Uni340F (CCTACGGGRBGCASCAG) and Bac806R (GGACTACYVGGGTATCTAAT) and reagents and conditions as previously described^51^. The first PCR reaction contained 1 × Q5 Hot Start High-Fidelity, 2 × Master Mix (New England Biolabs, Massachusetts, United States), 1 × EvaGreen Dye 20 × in water (Biotium, California, United States), 0.5 µM of each primer, and 3 µl of DNA in a final volume of 20 µL. In the amplification process, initial denaturation was performed at 98 ℃ for 30 s, followed by 35 cycles of denaturation at 98 ℃ for 15 s, annealing at 53 ℃ for 30 s and elongation at 72 ℃ for 20 s. The final elongation was performed at 72 ℃ for 10 min. The PCR product was purified with Agencourt AMPure XP beads (Beckman Coulter, Inc, Brea, CA, USA) according to the manufacturer’s instructions. The second PCR was performed with 4 µL of the purified PCR product to incorporate the adapters and the P5 and P7 Nextera indexes (Illumina, San Diego, CA, USA). The conditions for the second PCR were the same as above, except that the annealing temperature was 55 ℃, and 10 cycles were used. Libraries were cleaned and normalized using the SequalPrep Normalization Plate (96) Kit (ThermoFischer Scientific) and pooled together. Negative controls were included in the DNA extraction process (using only extraction kit reagents) and during library preparation (PCR-grade water). The library concentration was measured using Qubit 2 with the dsDNA HS kit (ThermoFischer Scientific), and the final sequencing was performed by Novogene on an Illumina NovaSeq platform with read lengths of 2 × 250 bp.

### Sequence analysis and statistical testing

Reads were quality filtered and trimmed using the Dada2 package using truncating of forward reads set to 260 bases and reverse reads set to 240 bases^70^. The error model in Dada2 was created using 1 million random filtered reads. Sequence variants (SV) were inferred using the Dada2 algorithm, and the removal of chimeras was performed using the function “removeBimeraDenovo” in the Dada2 R package. Sequence variants shorter than 375 base pairs were removed from the final table. Taxonomy was assigned using the Decipher R package^71^ against the SILVA SSU database^72^. Samples with less than 1500 reads and SVs with less than 10 sequences were removed from the table. For a few of the main interesting SVs, a search against the rRNA/ITS database at NCBI was performed using BLAST tool^73^. The name of the species with the highest % similarity was then retrieved and, in case two or more species had the same % similarity, all the species names were used. Alpha diversity was calculated using the Vegan package in R as described previously^3,74^. A pairwise comparison of the alpha diversity indexes between group levels was performed using the Kruskal–Wallis rank sum test. The alluvial plot was created with the Alluvial package in R.

### Identification of isolated bacteria with MALDI-TOF MS

The isolates that grew on TSA blood agar after inoculating milk samples in UHT milk with antimicrobial agents were identified using MALDI-TOF MS^75^. For this purpose, a colony of the isolate to be identified was transferred from TSA blood agar to a VITEK MS-DS slide (BioMerieux) and 1 µl VITEK MS CHCA matrix was applied on top. Ionization and mass analysis were done in the VITEK MS instrument with *E. coli* ATCC 8739 as a reference strain. The resulting peptide mass fingerprints were compared to the VITEK MS Expanded V3.2 Database and the results with a confidence value > 98% were considered identified.

### Minimal inhibitory concentration assays

Determination of MIC was performed on the isolates that were identified using MALDI-TOF MS. For this purpose, the broth microdilution method was utilized^21^. Briefly, a two-fold dilution series of penicillin G and AMC in 96 well microtiter plates with Hinton-Mueller broth were used. The dilutions used for both antimicrobial agents were (in µg/mL) 64, 32, 16, 8, 4, 2, 1, 0.5, 0.25, 0.125, 0.062. Wells without antimicrobial agents were included for each isolate. The microtiter plates were incubated at 37 ℃ in a Synergy H1 hybrid reader (BioTek) and OD_600_ was recorded every 10 min for 20 h. The MIC was recorded as the first well with severely reduced growth after 20 hours^22,23^.

### Supplementary Information

Supplementary Table S1.

## Data Availability

The 16S amplicon data is available in the EBI repository, https://www.ebi.ac.uk/, with accession number PRJEB70316.
