# Supplementary material for: An in vitro evaluation of the effect of antimicrobial treatment on bovine mammary microbiota
Source: Sci Rep. 2024 Aug 7;14:18333. doi: 10.1038/s41598-024-69273-y (PMC11306798; doi:10.1038/s41598-024-69273-y)
Supplement: Supplementary file 1 — Supplementary Table S1. [file 41598_2024_69273_MOESM1_ESM.docx]

**Title:** An *in vitro* evaluation of the effect of antimicrobial treatment on bovine mammary microbiota

Anja R. Winther^1,*^, Aurelie Perrin^1,¤^, Anne O. O. Nordraak^1,#^, Morten Kjos^1^, Davide Porcellato^1^

^1^ Faculty of Chemistry, Biotechnology and Food Science, The Norwegian University of Life Sciences, Ås, Norway.

^¤^ Current address: Institute Agro Dijon, 26 Bd Dr Petitjean, 21079 Dijon, France

^#^ Current address: Norwegian Defence Research Establishment, Kjeller, Norway

^*^ Corresponding author

**Table S1.** Overview of the minimal inhibitory concentration (MIC) using the broth microdilution method. The MIC for each isolate was determined for the antibiotic the isolate initially proliferated in. The MIC value was determined as the lowest concentration of the antibiotic required to completely inhibit bacterial growth after 20 hours.

| Amoxicillin/clavulanic acid | | | | |
| --- | --- | --- | --- | --- |
| **Sample** | **Species** | | **MIC (µg/mL)** | |
| H02 | *Enterococcus faecalis* | | 32 | |
| H02 | *Staphylococcus aureus* | | 16 | |
| H03 | *Corynebacterium amycolatum* | | 16 | |
| H05 | *Staphylococcus chromogenes* | | 16 | |
| H06 | *Staphylococcus epidermidis* | | 16 | |
| H08 | *Staphylococcus aureus* | | 32 | |
| H08 | *Staphylococcus simulans* | | 16 | |
| H08 | *Streptococcus dysgalactiae* spp. *equisimilis/dysgalactiae* | | 8 | |
| H09 | *Staphylococcus hominis* | | 32 | |
| H10 | *Staphylococcus haemolyticus* | | 16 | |
| H11 | *Staphylococcus chromogenes* | | 8 | |
| H12 | *Staphylococcus chromogenes* | | 32 | |
| H12 | *Staphylococcus saprophyticus* | | 32 | |
| H13 | *Staphylococcus xylosus* | | 32 | |
| H14 | *Micrococcu luteus* | | 16 | |
| H14 | *Staphylococcus saprophyticus* | | 32 | |
| H15 | *Staphylococcus epidermidis* | | 16 | |
| H17 | *Staphylococus saprophyticus* | | 32 | |
| H18 | *Corybacterium bovis* | | 16 | |
| H19 | *Staphylococcus cohnii* spp. *cohnii* | | 64 | |
| H21 | *Staphylococcus xylosus* | | 32 | |
| H22 | *Staphylococcus chromogenes* | | 16 | |
| H24 | *Staphylococcus epidermidis* | | >64 | |
| L01 | *Escherichia coli* | | >64 | |
| L01 | *Stenotrophomonas maltophilia* | | >64 | |
| L03 | *Enterococcus faecalis* | | 64 | |
| L03 | *Macrococcus caseolyticus* | | >64 | |
| L08 | *Staphylococcus xylosus* | | 64 | |
| L08 | *Streptococcus parasanguinis* | | >64 | |
| L17 | *Bacillus subtilis* | | 32 | |
| L20 | *Staphylococcus epidermidis* | | 32 | |
| L21 | *Pediococcus pentosaceus* | | >64 | |
|  |  | |  | |
| Penicillin G | | | | |
| **Sample** | | **Species** | | **MIC (µg/mL)** |
| H01 | | *Enterococcus durans* | | 64 |
| H01 | | *Escherichia coli* | | 64 |
| H02 | | *Enterococcus faecalis* | | 16 |
| H03 | | *Enhydrobacter aerosaccus/Moraxella osloensis* | | 0.063 |
| H03 | | *Micrococcu luteus* | | 0.125 |
| H05 | | *Micrococcu luteus* | | 0.125 |
| H06 | | *Staphylococcus epidermidis* | | >64 |
| H08 | | *Staphylococcus aureus* | | 0.125 |
| H10 | | *Bacillus cereus* group | | >64 |
| H10 | | *Corynebacterium amycolatum* | | 64 |
| H10 | | *Staphylococcus epidermidis* | | >64 |
| H10 | | *Staphylococcus epidimidis* | | 64 |
| H13 | | *Corybacterium bovis* | | 8 |
| H15 | | *Staphylococcus epidermidis* | | >64 |
| H23 | | *Staphylococcus epidermidis* | | >64 |
| H24 | | *Staphylococcus epidermidis* | | >64 |
| H25 | | *Kocuria rhizophila* | | >64 |
| H25 | | *Staphylococcus epidermidis* | | >64 |
| H25 | | *Weissella paramesenteroides* | | 4 |
| L01 | | *Enterococcus faecalis* | | 16 |
| L03 | | *Enterococcus faecalis* | | 8 |
| L03 | | *Enterococcus faecium* | | 4 |
| L05 | | *Escherichia coli* | | >64 |
| L10 | | *Micrococcu luteus* | | 0.125 |
| L16 | | *Staphylococcus epidermidis* | | 8 |
| L21 | | *Staphylococcus epidermidis* | | 0.062 |
